# HIF-PH Inhibitor Promotes Stabilization of HIF-1α via Inhibition of Its Degradation and Exerts Chondroprotective Effects in a Rat Osteoarthritis Model

**DOI:** 10.3390/ijms27177869

**Published:** 2026-09-03

**Authors:** Kei Nakamura, Yuta Fujii, Yuji Arai, Shuji Nakagawa, Atsuo Inoue, Ryota Cha, Keisuke Sugie, Kentaro Hayashi, Tomoki Saito, Tsunao Kishida, Osam Mazda, Kenji Takahashi

**Affiliations:** 1 Department of Orthopaedics, Graduate School of Medical Science, Kyoto Prefectural University of Medicine, Kawaramachi-Hirokoji, Kamigyo-ku, Kyoto 602-8566, Japan; keinaka2@koto.kpu-m.ac.jp (K.N.); y-fujii@koto.kpu-m.ac.jp (Y.F.); a-inoue@koto.kpu-m.ac.jp (A.I.); r-cha@koto.kpu-m.ac.jp (R.C.); k-sugie@koto.kpu-m.ac.jp (K.S.); aparagi@koto.kpu-m.ac.jp (K.H.); saitot@koto.kpu-m.ac.jp (T.S.); kenji-am@koto.kpu-m.ac.jp (K.T.); 2 Department of Sports and Para-Sports Medicine, Graduate School of Medical Science, Kyoto Prefectural University of Medicine, Kawaramachi-Hirokoji, Kamigyo-ku, Kyoto 602-8566, Japan; shushi@koto.kpu-m.ac.jp; 3 Department of Immunology, Graduate School of Medical Science, Kyoto Prefectural University of Medicine, Kawaramachi-Hirokoji, Kamigyo-ku, Kyoto 602-8566, Japan; tsunao@koto.kpu-m.ac.jp (T.K.); mazda@koto.kpu-m.ac.jp (O.M.)

**Keywords:** HIF-PH inhibitor, HIF-1α, osteoarthritis

## Abstract

Articular cartilage exists under hypoxic conditions, where hypoxia-inducible factor (HIF)-1α plays a critical role in maintaining its homeostasis. In osteoarthritis (OA), however, this hypoxic environment is disrupted, and decreased HIF-1α expression contributes to disease progression. HIF-prolyl hydroxylase (HIF-PH) inhibitors stabilize HIF-1α and are clinically used to treat renal anemia; therefore, they may also exert therapeutic effects in OA through the same mechanism. However, their effects on articular cartilage remain unclear. In this study, we investigated the effects of Roxadustat, a HIF-PH inhibitor, both in vitro using rat chondrocytes and in vivo using a monosodium iodoacetate (MIA)-induced rat OA model. Roxadustat showed no cytotoxicity and significantly increased the protein expression of HIF-1α, SRY-box transcription factor 9 (SOX9), and Aggrecan in monolayer cultures. In three-dimensional spheroid cultures, Roxadustat enhanced Safranin O staining and extracellular matrix production and significantly upregulated SOX9 and ACAN mRNA expression. Furthermore, intra-articular administration of Roxadustat in the MIA-induced OA model suppressed cartilage degeneration and significantly reduced the Modified Mankin score. These findings demonstrate that Roxadustat promotes anabolic responses in chondrocytes through stabilization of HIF-1α and suppresses cartilage degeneration in OA. Intra-articular administration of HIF-PH inhibitors may represent a novel disease-modifying therapeutic strategy for OA.

## 1. Introduction

Osteoarthritis (OA) is a chronic progressive disease characterized by degeneration and dysfunction of articular cartilage associated with aging [1]. Currently, no treatment exists that can fundamentally prevent OA progression; therefore, the development of disease-modifying osteoarthritis drugs (DMOADs) is an urgent unmet need [2,3]. Articular cartilage is an avascular tissue, and chondrocytes are physiologically adapted to hypoxic environments [4,5,6]. Hypoxia-inducible factor (HIF)-1α is a key transcription factor that mediates cellular responses to hypoxia. It regulates the expression of anabolic genes involved in cartilage matrix synthesis and plays a crucial role in maintaining cartilage homeostasis [7,8]. Previous studies have shown that deficiency of HIF-1α reduces cartilage matrix production [9] and that HIF-1α signaling is attenuated with the progression of OA [10]. The role of HIF-1α in OA appears to be highly context-dependent and may differ according to the cell type and joint tissue involved. In articular cartilage, HIF-1α is essential for chondrocyte survival, metabolic adaptation to the hypoxic environment, and maintenance of cartilage homeostasis through regulation of anabolic gene expression. In contrast, increased HIF-1α activity has been reported in OA synovial fibroblasts, where it may promote inflammatory responses and angiogenesis. These findings suggest that the biological effects of HIF-1α are tissue-specific rather than uniformly protective or detrimental in OA. Therefore, the present study focuses specifically on the role of HIF-1α stabilization in articular cartilage and its potential chondroprotective effects. These findings suggest that the accumulation and stabilization of HIF-1α may represent a novel therapeutic strategy to suppress cartilage degeneration. However, HIF-1α is tightly regulated by oxygen-dependent degradation via prolyl hydroxylase domain-containing protein (PHD), making it difficult to maintain stable expression [11]. HIF-PH inhibitors, which suppress PHD activity and stabilize HIF signaling, are currently used clinically for the treatment of renal anemia in patients with chronic kidney disease [12,13]. Since the first global approval of Roxadustat, a HIF-PH inhibitor, in China in 2018, various HIF-PH inhibitors, including Roxadustat, have been clinically used worldwide [14,15]. Drug repositioning, the strategy of applying approved drugs to new indications, offers advantages in terms of safety and development time. We hypothesized that stabilization of HIF-1α in articular cartilage using HIF-PH inhibitors could enhance anabolic responses and represent a novel therapeutic approach for OA. Therefore, the aim of this study was to evaluate the effects of the clinically approved HIF-PH inhibitor Roxadustat on articular cartilage and to investigate its chondroprotective effects via HIF-1α stabilization using both in vitro and in vivo approaches.

## 2. Results

### 2.1. Effects of Roxadustat on Cell Viability in Rat Chondrocytes

**Figure 1:**
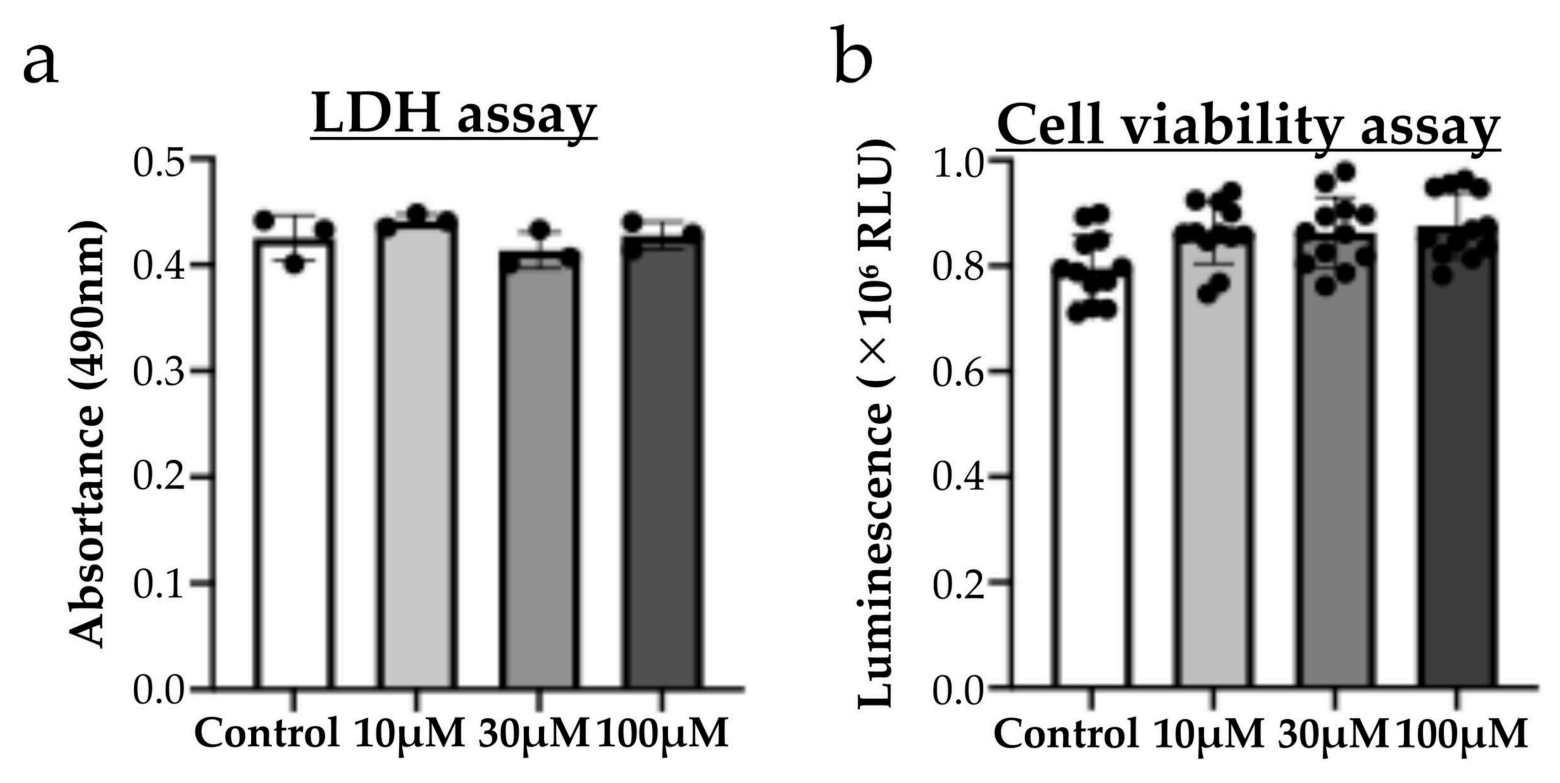
Cytotoxicity and cell viability of Roxadustat in rat chondrocyte monolayer cultures after 48 h of treatment. (**a**) Cytotoxicity was assessed by LDH assay (n = 3 per group). (**b**) Cell viability was assessed by the RealTime-Glo™ MT Cell Viability Assay (n = 12 per group).

Roxadustat (Selleck Biotech, Tokyo, Japan) was used as the HIF-PH inhibitor in this study. Cytotoxicity of Roxadustat in rat chondrocytes was evaluated using an LDH assay. No significant differences in LDH release were observed between the control group and groups treated with 10, 30, or 100 μM Roxadustat, indicating that Roxadustat did not induce cytotoxicity under the experimental conditions (Figure 1a). In addition, cell viability was assessed using the RealTime-Glo™ MT Cell Viability Assay (Promega, Madison, WI, USA). No significant differences in luminescence intensity were observed between the control group and groups treated with 10, 30, or 100 μM Roxadustat, indicating that Roxadustat did not adversely affect chondrocyte viability (Figure 1b). These findings demonstrate that Roxadustat does not exhibit cytotoxicity and does not impair the viability of rat chondrocytes under the present experimental conditions.

### 2.2. Effects of Roxadustat on Metabolism-Related Factors in Rat Chondrocytes

**Figure 2:**
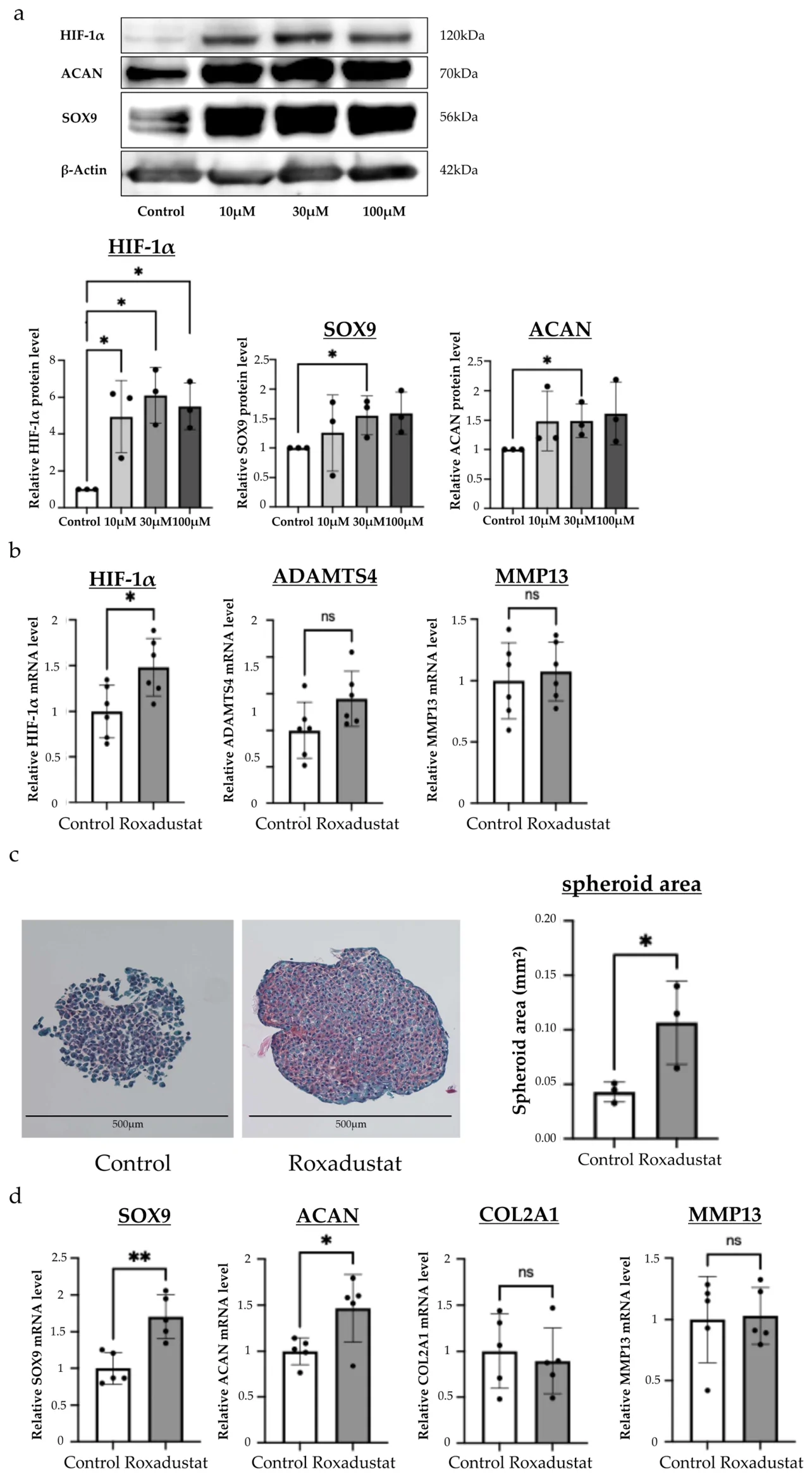
(**a**) Western blot analysis of monolayer cultures after 48 h of Roxadustat treatment (n = 3 per group). (**b**) Real-time RT-PCR analysis of monolayer cultures after 48 h of Roxadustat treatment (n = 6 per group). (**c**) Safranin O staining of spheroid sections after 14 days of Roxadustat treatment (scale bar = 500 μm) and quantification of spheroid area (mean ± SEM) (n = 3 per group). (**d**) Real-time RT-PCR analysis of spheroid cultures after 14 days of Roxadustat treatment (n = 5 per group). * *p* < 0.05, ** *p* < 0.01.

To evaluate the effects of Roxadustat on rat chondrocytes, protein expression was first analyzed in monolayer cultures. Western blot analysis demonstrated that treatment with 30 μM Roxadustat significantly increased the expression of HIF-1α, SOX9, and Aggrecan compared with the control group (Figure 2a). In addition, real-time RT-PCR analysis of monolayer cultures demonstrated that HIF-1α mRNA expression was significantly upregulated by Roxadustat treatment. In contrast, the expression levels of the catabolic markers ADAMTS4 and MMP13 were not significantly different between the Roxadustat-treated and control groups (Figure 2b).

Next, histological evaluation and analysis of cartilage matrix-related gene expression were performed in three-dimensional spheroid cultures. Based on the results of the monolayer culture experiments, the concentration of Roxadustat was set at 30 μM. Histological evaluation using Safranin O staining demonstrated enhanced Safranin O staining intensity in spheroids of the Roxadustat-treated group. Furthermore, spheroid area (mm^2^) was significantly greater in the Roxadustat group (0.11 ± 0.04) than in the control group (0.04 ± 0.01) (*p* < 0.05), indicating increased extracellular matrix production (Figure 2c). Real-time RT-PCR analysis showed significant upregulation of anabolic markers SOX9 and ACAN in the Roxadustat group compared with the control group. In contrast, no significant differences were observed in COL2A1 or the catabolic marker MMP13 between the two groups (Figure 2d).

### 2.3. Effects of Intra-Articular Roxadustat Administration in MIA-Induced OA Rats

**Figure 3:**
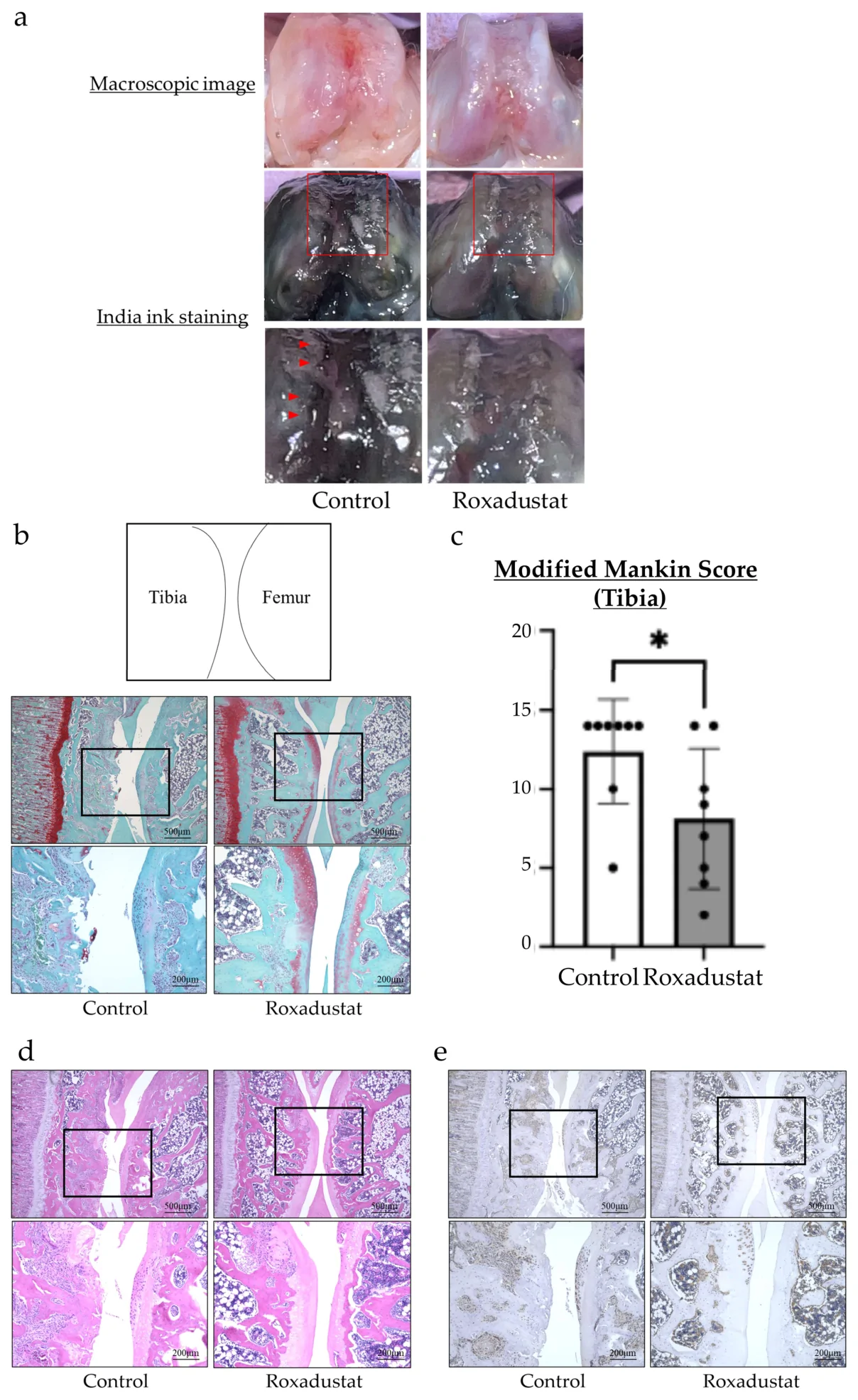
(**a**) Representative macroscopic and India ink–stained images after 28 days of Roxadustat administration (▼; cartilage lesion). (**b**) Representative sagittal sections stained with Safranin O staining (scale bars = 500 μm in the upper panels and 200 μm in the lower panels). (**c**) Modified Mankin score (mean ± SEM) (n = 8 per group). (**d**) Representative sagittal sections stained with hematoxylin and eosin (H&E) staining (scale bars = 500 μm in the upper panels and 200 μm in the lower panels). (**e**) Representative sagittal sections stained with HIF-1α immunohistochemical staining (scale bars = 500 μm in the upper panels and 200 μm in the lower panels). * *p* < 0.05.

To evaluate the effects of Roxadustat in vivo, Roxadustat was administered intra-articularly in a rat OA model induced by monosodium iodoacetate (MIA; Sigma-Aldrich, St. Louis, MO, USA). Based on in vitro findings, the concentration of Roxadustat was set at 30 μM. Macroscopic evaluation using India ink staining showed irregular cartilage surfaces and extensive lesions in the control group, whereas the Roxadustat group exhibited smoother cartilage surfaces with smaller areas of degeneration (Figure 3a). Histological evaluation using Safranin O staining demonstrated marked loss of cartilage matrix and reduced Safranin O staining intensity in the control group, whereas cartilage degeneration was suppressed in the Roxadustat group (Figure 3b). Quantitative evaluation using the Modified Mankin score revealed significantly lower scores in the Roxadustat group (8.13 ± 4.45) compared with the control group (12.38 ± 3.30) (*p* < 0.05) (Figure 3c). The effect size for the difference in Modified Mankin score between the two groups was large (Cohen’s d = 1.09). Hematoxylin and eosin (H&E) staining further confirmed the preservation of cartilage architecture and reduced cartilage degeneration in the Roxadustat group compared with the control group (Figure 3d). In addition, immunohistochemical staining demonstrated increased HIF-1α expression in the articular chondrocytes of the Roxadustat group (Figure 3e).

## 3. Discussion

The principal finding of this study is that the HIF-PH inhibitor Roxadustat exerts chondroprotective effects in both rat chondrocytes and an MIA-induced OA model.

HIF-1α plays a crucial role in hypoxic adaptation in chondrocytes [11,16], and its stabilization contributes to the maintenance of cellular phenotype [10]. In chondrocytes, HIF-1α is known to be involved not only in matrix production but also in the regulation of intracellular metabolic reprogramming and cell survival signaling [16,17]. Chondrocytes maintain their function by relying on glycolytic metabolism under hypoxic conditions, and stabilization of HIF-1α may support this metabolic adaptation [16]. Furthermore, HIF-1α has been associated with stress responses and autophagy activation [5]. Roxadustat inhibits HIF degradation by competitively binding to the active site of PHD, thereby stabilizing HIF expression [18]. Ughetta et al. reported that Roxadustat increases HIF-1α protein expression in a dose-dependent manner in Hep3B and HK-2 cells [13], whereas Browe et al. demonstrated that Roxadustat promotes chondrogenic differentiation of mesenchymal stem cells via stabilization of HIF signaling [19]. However, its effects on chondrocyte homeostasis have not been fully elucidated.

In this study, Roxadustat increased intracellular accumulation of HIF-1α and significantly enhanced the expression of selected anabolic factors, including SOX9 and Aggrecan, without significantly increasing catabolic factors, including Mmp13 and Adamts4, or adversely affecting cell viability. These findings suggest that Roxadustat may promote an anabolic response in chondrocytes without concomitant activation of the catabolic factors examined in this study. In chondrocytes, HIF-1α has been reported to regulate the transcriptional activity of SOX9 and to directly and indirectly modulate the expression of cartilage matrix–related genes, including COL2A1 and ACAN [7,8]. The observed increase in matrix production in this study may be associated with enhanced anabolic activity following HIF-1α stabilization. Although SOX9 expression was significantly increased, COL2A1 expression was not significantly altered, suggesting that the anabolic effects of Roxadustat may involve regulatory mechanisms beyond the HIF-1α–SOX9 axis. Furthermore, the observed increase in Safranin O staining intensity and matrix area in the three-dimensional culture system is consistent with enhanced cartilage matrix formation following Roxadustat treatment under conditions that more closely mimic the in vivo environment. Although the present study did not directly investigate pathways involved in intracellular metabolic reprogramming or regulation of cell survival signaling, HIF-1α stabilization induced by Roxadustat may have contributed not only to enhanced matrix synthesis but also to the maintenance of chondrocyte function and improvement of the cellular microenvironment.

In vivo, intra-articular administration of Roxadustat in the MIA-induced OA model significantly suppressed the progression of cartilage degeneration, as demonstrated by both macroscopic and histological findings, and resulted in a significantly lower Modified Mankin score. Although the MIA model induces rapid chondrocyte injury through inhibition of glycolysis, histological and immunohistochemical findings demonstrated preserved chondrocytes with HIF-1α expression following Roxadustat treatment. These findings suggest that HIF-1α stabilization is associated with adaptive and anabolic responses in surviving chondrocytes. However, the present findings do not exclude the contribution of additional HIF-dependent or HIF-independent signaling pathways to the observed chondroprotective effects. Li et al. reported that oral administration of Roxadustat suppressed bone loss in an ovariectomized (OVX) rat model through accumulation of HIF-1α in bone tissue [20], whereas Zabura et al. demonstrated that intraperitoneal administration of Roxadustat attenuated myocardial remodeling in a mouse model of myocardial infarction via HIF-1α accumulation [21]. These findings suggest that Roxadustat exerts tissue-protective effects across various tissues through stabilization of HIF-1α. However, its effects on articular cartilage have not been previously reported, and this study is the first to demonstrate its potential efficacy in this context.

Furthermore, while Roxadustat has traditionally been administered systemically, this study is characterized by the use of intra-articular local administration. Local delivery offers the advantage of reducing the risk of systemic adverse effects while enabling higher drug concentrations at the target tissue. These findings suggest that local stabilization of HIF-1α signaling using an approved drug may represent a promising disease-modifying therapeutic strategy for OA.

This study has several limitations. First, the MIA-induced OA model used in this study is a chemically induced OA model that primarily evaluates protective effects on articular cartilage. Therefore, further validation using animal models that more closely mimic the clinical progression of OA, such as load-induced OA models, is warranted. Second, only Roxadustat was examined among HIF-PH inhibitors, and only a single dosing regimen was evaluated. Further studies using other HIF-PH inhibitors and different administration protocols are needed. Third, although this study demonstrated HIF-1α stabilization and enhanced anabolic responses, the underlying molecular mechanisms were not fully elucidated. We did not directly evaluate metabolic alterations, early chondrocyte viability following MIA injection, apoptosis, or proliferation. Although chondrocyte viability was confirmed using both CCK-8 and RealTime-Glo™ MT Cell Viability assays, further studies using metabolic analyses, flow cytometry, EdU staining, and comprehensive transcriptomic approaches such as RNA sequencing are warranted to clarify the mechanisms underlying the chondroprotective effects of Roxadustat. In addition, catabolic factors were evaluated only at the mRNA level, and protein-level validation, including Western blot analysis of MMPs, was not performed. Further studies are needed to confirm these findings at the protein level. Fourth, the dose of intra-articular Roxadustat was selected based on the effective concentration identified in vitro rather than on pharmacokinetic or pharmacodynamic data. Further studies evaluating intra-articular pharmacokinetics, dose–response relationships, and optimal dosing regimens are required. Fifth, an a priori sample size calculation was not performed. Although a statistically significant difference with a large effect size was observed, the relatively large variability in the histological scores suggests that studies with larger sample sizes are warranted to confirm the reproducibility of these findings. Finally, this study did not determine whether the protective effects of Roxadustat were mediated exclusively through HIF-1α stabilization or also involved HIF-independent mechanisms. Therefore, we cannot exclude the possibility that additional HIF-dependent or HIF-independent pathways contribute to the observed chondroprotective effects. Future studies using genetic or pharmacological approaches to selectively manipulate HIF-1α signaling will help clarify the precise mechanisms of action.

## 4. Materials and Methods

### 4.1. In Vitro Studies

#### 4.1.1. Isolation and Culture of Rat Articular Chondrocytes

Four 6-week-old male Wistar rats (Shimizu Laboratory Suppliers, Kyoto, Japan) were deeply anesthetized with pentobarbital and euthanized. Articular cartilage was aseptically harvested from the knee, hip, and shoulder joints. The cartilage tissue was minced and digested enzymatically to isolate chondrocytes, which were then seeded into 75-cm^2^ culture flasks (Corning Incorporated, Corning, NY, USA). Cells were cultured at 37 °C in a humidified atmosphere of 5% CO_2_ and 95% air (standard conditions) using Dulbecco’s modified Eagle’s medium (DMEM; Nacalai Tesque, Kyoto, Japan) supplemented with 10% fetal bovine serum (Biosera, Cholet, France) and 1% penicillin–streptomycin solution (Nacalai Tesque, Kyoto, Japan). All in vitro experiments were performed using primary (P0) rat articular chondrocytes.

#### 4.1.2. Monolayer Culture Experiments

For monolayer culture, chondrocytes were seeded at a density of 1 × 10^5^ cells per 35 mm dish (AGC Techno Glass, Shizuoka, Japan). When the cells reached approximately 70–80% confluence, they were divided into the Control group and the Roxadustat-treated group. Roxadustat was dissolved in dimethyl sulfoxide (DMSO; Wako Pure Chemical Industries, Osaka, Japan) and added to the treated group at concentrations of 10, 30, and 100 µM, while the same volume of DMSO was added to the Control group. Cells were then incubated for 48 h under identical culture conditions (37 °C, 5% CO_2_, humidified atmosphere).

#### 4.1.3. Spheroid Culture Experiments

For spheroid culture, rat articular chondrocytes were seeded at a density of 1 × 10^5^ cells per well in 96-well U-bottom plates (Sumitomo Bakelite Co., Ltd., Tokyo, Japan) and centrifuged at 1500 rpm for 10 min to promote cell aggregation. After spheroid formation, the cells were divided into two groups: a Control group and a Roxadustat-treated group. Roxadustat was dissolved in DMSO and added to the treated group at a concentration of 30 µM, while the same volume of DMSO was added to the Control group. The spheroids were cultured for 14 days under identical conditions (37 °C, 5% CO_2_, humidified atmosphere).

#### 4.1.4. Lactate Dehydrogenase (LDH) Release Assay

After 48 h of treatment in monolayer cultures, cytotoxicity was evaluated using the LDH Cytotoxicity Assay Kit (Nacalai Tesque, Kyoto, Japan) according to the manufacturer’s instructions. Briefly, 100 µL of culture supernatant was collected from each well and mixed with 100 µL of Substrate Solution. After incubation for 20 min under normoxic conditions in the dark, the reaction was terminated by adding 50 µL of Stop Solution. The optical density (OD) was then measured spectrophotometrically at 490 nm.

#### 4.1.5. RealTime-Glo™ MT Cell Viability Assay

After 48 h of treatment in monolayer cultures, cell viability was evaluated using the RealTime-Glo™ MT Cell Viability Assay (Promega, Madison, WI, USA) according to the manufacturer’s instructions. Briefly, the RealTime-Glo™ MT Cell Viability Substrate and Enzyme were prepared according to the manufacturer’s protocol and added directly to the culture medium. Luminescence was measured using a microplate luminometer after incubation under standard culture conditions. Luminescence intensity was used as an indicator of metabolically active viable cells.

#### 4.1.6. Real-Time Reverse Transcription Polymerase Chain Reaction (RT-PCR)

For monolayer cultures (n = 6), rat chondrocytes were treated with 30 μM Roxadustat for 48 h, after which total RNA was extracted using ISOGEN II (Nippon Gene, Osaka, Japan) according to the manufacturer’s instructions. Reverse transcription was performed using ReverTra Ace^®^ qPCR RT Master Mix (Toyobo, Osaka, Japan) to synthesize complementary DNA (cDNA). Quantitative real-time RT-PCR was conducted using the Applied Biosystems 7300 Real-Time PCR System (Applied Biosystems, Carlsbad, CA, USA) with TaqMan gene expression assays (Applied Biosystems) specific for Hif1a (Rn01472831_m1), Adamts4 (Rn01490214_m1), and Mmp13 (Rn01448185_g1).

For spheroid cultures (n = 5), total RNA was extracted after 14 days of culture using ISOGEN II according to the manufacturer’s instructions. Reverse transcription and quantitative real-time RT-PCR were performed using the same procedure as described above with TaqMan gene expression assays specific for Col2a1 (Rn01637087_m1), Acan (Rn00573424_m1), Sox9 (Rn01751070_m1), and Mmp13 (Rn01448185_g1).

Each 20 µL reaction mixture contained 2 µL of cDNA and 10 µL of TaqMan^®^ Gene Expression PCR Master Mix (Toyobo, Osaka, Japan) for the target gene. The amplification protocol consisted of 40 cycles of denaturation at 95 °C for 15 s and annealing/extension at 60 °C for 1 min. Relative gene expression levels were calculated using the 2^−ΔΔCt^ method. The 18S ribosomal RNA was used as the internal control [forward primer, 5′-ATGAGTCCACTTTAAATCCTTTAACGA-3′; reverse primer, 5′-CTTTAATATACGCTATTGGAGCTGGAA-3′; probes, 5′-(FAM)ATCCATTGGAGGGCAAGTCTGGTGC(BHQ)-3′].

#### 4.1.7. Western Blot Analysis

After 48 h of treatment in monolayer cultures, proteins were collected for Western blot analysis. Cells were scraped and lysed in 150 µL of RIPA buffer (Nacalai Tesque, Kyoto, Japan). Lysates were centrifuged at 15,000 rpm for 5 min, and the supernatants were collected. Protein concentrations were determined using the bicinchoninic acid (BCA) assay, and equal amounts of total protein were subjected to sodium dodecyl sulfate polyacrylamide gel electrophoresis (SDS-PAGE). Samples containing 5.0 µg of protein were separated on 10% Bis-Tris NuPAGE^®^ gels (Thermo Fisher Scientific, Waltham, MA, USA) using 5% MOPS SDS running buffer. Proteins were transferred onto PVDF membranes by wet blotting. Membranes were blocked with Blocking One solution (Nacalai Tesque, Kyoto, Japan) for 30 min at room temperature and then incubated overnight at 4 °C with primary antibodies (1:1000 dilution): HIF-1α (ab179483, Abcam, Cambridge, UK), SOX9 (ab185966, Abcam, Cambridge, UK), and Aggrecan (ab3778, Abcam). β-actin (A5316, Sigma-Aldrich, St. Louis, MO, USA) was used as a loading control. After washing the membranes two to three times with TBST, peroxidase-conjugated secondary antibodies (1:6000 dilution; anti-mouse IgG [A4416, Sigma-Aldrich, St. Louis, MO, USA] and anti-rabbit IgG [A0545, Sigma-Aldrich]) were applied for 1 h at room temperature. The blots were washed again with TBST, and chemiluminescent signals were detected using Chemi-Lumi One Super (Nacalai Tesque) and visualized with an imaging system. Protein band intensities were quantified using ImageJ software (version 1.54g, NIH, Bethesda, MD, USA) and normalized to β-actin. The normalized values were used for quantitative analysis and are presented as relative protein expression levels.

#### 4.1.8. Histological Evaluation of Spheroids

After 14 days of culture, spheroids (n = 3) were collected and fixed in 4% paraformaldehyde (PFA; Nacalai Tesque, Kyoto, Japan) for 24 h at 4 °C. The fixed spheroids were embedded in paraffin, sectioned at a thickness of 5 µm, and stained with Safranin O to evaluate cartilage matrix formation. The stained sections were observed under an all-in-one fluorescence microscope (BZ-X series; Keyence, Osaka, Japan).

### 4.2. In Vivo Studies

#### 4.2.1. Animals

The experiment was conducted using a total of sixteen 8-week-old male Wistar rats. All animal procedures were performed in accordance with the institutional guidelines for animal experimentation and were approved by the Animal Research Committee of our institution (Approval Code: M2024-259).

#### 4.2.2. Rat OA Model

An OA model was established by intra-articular injection of 1 mg of MIA dissolved in 50 µL of phosphate-buffered saline (PBS) into the left knee joint of eight-week-old male Wistar rats.

#### 4.2.3. Roxadustat Treatment

**Figure 4:**
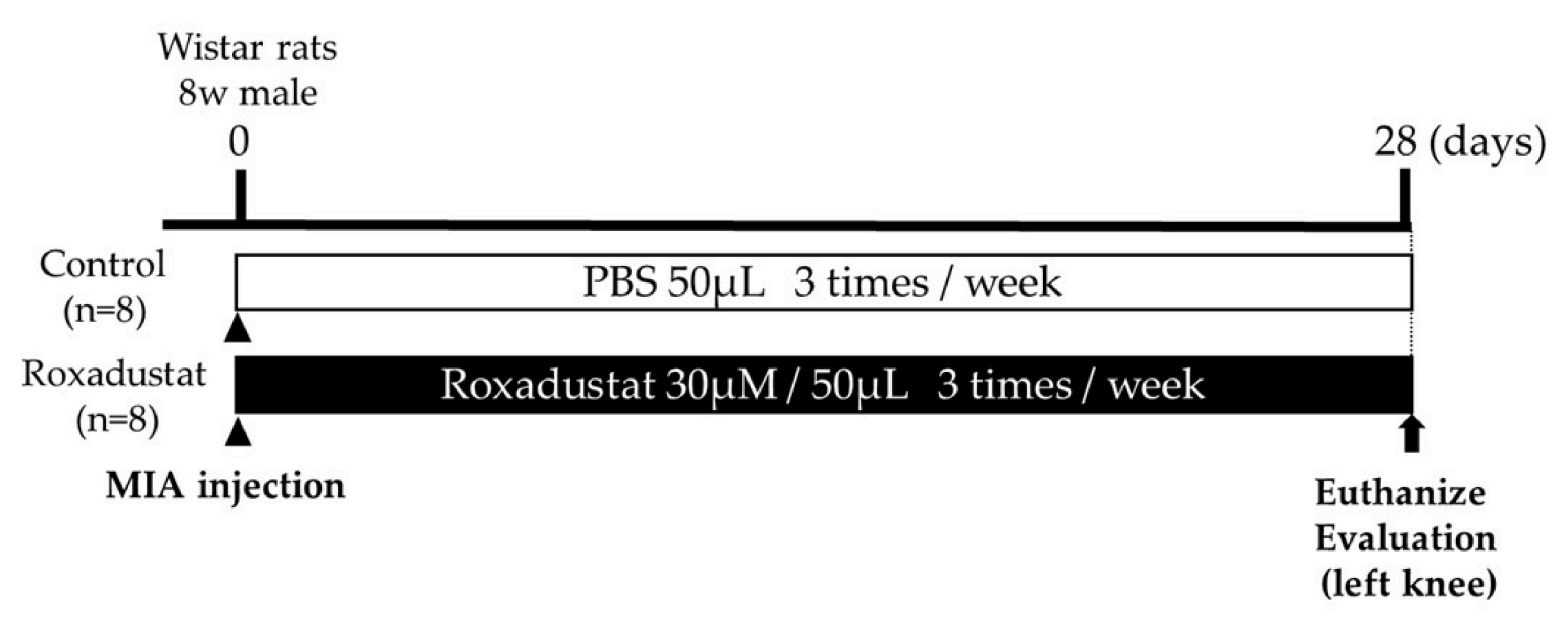
Experimental protocol for evaluation of Roxadustat treatment in MIA-induced osteoarthritic rats.

On the day after OA induction with MIA, the rats were randomly divided into two groups: the Control group and the Roxadustat-treated group. In the Roxadustat group, Roxadustat was dissolved in PBS at a concentration of 30 µM (approximately 0.53 µg per 50 µL injection). In the Control group, an equal amount of DMSO was dissolved in PBS. Each rat received 50 µL of either the Roxadustat solution or the control solution injected into the left knee joint three times per week, beginning the day after MIA injection. The concentration of Roxadustat was selected based on the effective concentration identified in the in vitro experiments, and the injection volume (50 µL) was determined according to the capacity of the rat knee joint. The left knee joints were harvested 28 days after the start of treatment (Figure 4).

#### 4.2.4. Histological Analysis

Both groups of rats were euthanized 28 days after MIA injection. The left knee joints were harvested, and cartilage degeneration sites were identified by India ink staining. The specimens were fixed in 4% paraformaldehyde (Wako, Osaka, Japan), decalcified with 20% ethylenediaminetetraacetic acid (EDTA), and embedded in paraffin. Sagittal sections with a thickness of 6 µm were prepared through the central region of the medial femoral condyle and stained with Safranin O and hematoxylin and eosin (H&E). Semiquantitative histopathological evaluation was performed using the Modified Mankin scoring system [22,23]. Immunohistochemical staining for HIF-1α was performed using an anti-HIF-1α antibody (ab114977; Abcam, Cambridge, UK; 1:100 dilution). Immunoreactivity was visualized with 3,3′-diaminobenzidine (DAB), and the sections were counterstained with hematoxylin.

### 4.3. Statistical Analysis

Data are presented as the mean ± standard error of the mean (SEM). Statistical analyses were performed using EZR (Saitama Medical Center, Jichi Medical University, Saitama, Japan), which is a graphical user interface for R (The R Foundation for Statistical Computing, Vienna, Austria). Normality of data distribution was assessed using the Shapiro–Wilk test prior to statistical analysis. Comparisons between two groups were analyzed using Student’s *t*-test. In the monolayer experiments, each Roxadustat concentration (10, 30, and 100 µM) was compared with the Control group using independent two-tailed Student’s *t*-tests. Because the objective was to compare each concentration with the Control group, no comparisons were made among Roxadustat concentrations. *p* < 0.05 was considered to indicate a statistically significant difference.

## 5. Conclusions

This study demonstrates that the HIF-PH inhibitor Roxadustat promotes anabolic responses in chondrocytes through stabilization of HIF-1α. Furthermore, Roxadustat significantly suppresses cartilage degeneration in an MIA-induced OA rat model. These findings suggest that HIF-PH inhibitors may represent a novel disease-modifying therapeutic strategy for OA.

## Data Availability

The datasets used and/or analyzed during the current study are available from the corresponding author on reasonable request.

## References

[1] Martel-Pelletier J., Pelletier J.P. (2025). Next-Level Prediction of Structural Progression in Knee Osteoarthritis: A Perspective. Int. J. Mol. Sci..

[2] Molnar V., Matišić V., Kodvanj I., Bjelica R., Jeleč Ž., Hudetz D., Rod E., Čukelj F., Vrdoljak T., Vidović D. (2021). Cytokines and Chemokines Involved in Osteoarthritis Pathogenesis. Int. J. Mol. Sci..

[3] Kim E.H., Jeon S., Park J., Ryu J.H., Mobasheri A., Matta C., Jin E.J. (2025). Progressing future osteoarthritis treatment toward precision medicine: Integrating regenerative medicine, gene therapy and circadian biology. Exp. Mol. Med..

[4] Liu Y., Shah K.M., Luo J. (2021). Strategies for Articular Cartilage Repair and Regeneration. Front. Bioeng. Biotechnol..

[5] Lu J., Peng Y., Zou J., Wang J., Lu S., Fu T., Jiang L., Zhang C., Zhang J. (2021). Hypoxia Inducible Factor-1α Is a Regulator of Autophagy in Osteoarthritic Chondrocytes. Cartilage.

[6] Lammi M.J., Qu C. (2024). Regulation of Oxygen Tension as a Strategy to Control Chondrocytic Phenotype for Cartilage Tissue Engineering and Regeneration. Bioengineering.

[7] Zhang K., Fu W. (2024). HIF-1*α*: Linking subchondral bone and cartilage as a therapeutic target in osteoarthritis. Biomater. Transl..

[8] Zhang X.A., Kong H. (2023). Mechanism of HIFs in osteoarthritis. Front. Immunol..

[9] Pfander D., Cramer T., Schipani E., Johnson R.S. (2003). HIF-1alpha controls extracellular matrix synthesis by epiphyseal chondrocytes. J. Cell Sci..

[10] Zhang H., Wang L., Cui J., Wang S., Han Y., Shao H., Wang C., Hu Y., Li X., Zhou Q. (2023). Maintaining hypoxia environment of subchondral bone alleviates osteoarthritis progression. Sci. Adv..

[11] Cha R., Nakagawa S., Arai Y., Inoue A., Okubo N., Fujii Y., Kaihara K., Nakamura K., Kishida T., Mazda O. (2025). Intermittent hypoxic stimulation promotes efficient expression of Hypoxia-inducible factor-1α and exerts a chondroprotective effect in an animal osteoarthritis model. PLoS ONE.

[12] Bartnicki P. (2024). Hypoxia-Inducible Factor Prolyl Hydroxylase Inhibitors as a New Treatment Option for Anemia in Chronic Kidney Disease. Biomedicines.

[13] Del Balzo U., Signore P.E., Walkinshaw G., Seeley T.W., Brenner M.C., Wang Q., Guo G., Arend M.P., Flippin L.A., Chow F.A. (2020). Nonclinical Characterization of the Hypoxia-Inducible Factor Prolyl Hydroxylase Inhibitor Roxadustat, a Novel Treatment of Anemia of Chronic Kidney Disease. J. Pharmacol. Exp. Ther..

[14] Dhillon S. (2019). Roxadustat: First Global Approval. Drugs.

[15] Chen N., Hao C., Peng X., Lin H., Yin A., Hao L., Tao Y., Liang X., Liu Z., Xing C. (2019). Roxadustat for Anemia in Patients with Kidney Disease Not Receiving Dialysis. N. Engl. J. Med..

[16] Zeng C.Y., Wang X.F., Hua F.Z. (2022). HIF-1α in Osteoarthritis: From Pathogenesis to Therapeutic Implications. Front. Pharmacol..

[17] Basheeruddin M., Qausain S. (2024). Hypoxia-Inducible Factor 1-Alpha (HIF-1α): An Essential Regulator in Cellular Metabolic Control. Cureus.

[18] Liang Q., Li X., Niu Q., Zhao H., Zuo L. (2023). Efficacy and Safety of Roxadustat in Chinese Hemodialysis Patients: A Systematic Review and Meta-Analysis. J. Clin. Med..

[19] Browe D.C., Coleman C.M., Barry F.P., Elliman S.J. (2019). Hypoxia Activates the PTHrP -MEF2C Pathway to Attenuate Hypertrophy in Mesenchymal Stem Cell Derived Cartilage. Sci. Rep..

[20] Li L., Li A., Zhu L., Gan L., Zuo L. (2022). Roxadustat promotes osteoblast differentiation and prevents estrogen deficiency-induced bone loss by stabilizing HIF-1α and activating the Wnt/β-catenin signaling pathway. J. Orthop. Surg. Res..

[21] Zaruba M.M., Staggl S., Ghadge S.K., Maurer T., Gavranovic-Novakovic J., Jeyakumar V., Schönherr P., Wimmer A., Pölzl G., Bauer A. (2024). Roxadustat Attenuates Adverse Remodeling Following Myocardial Infarction in Mice. Cells.

[22] Muehleman C., Bareither D., Huch K., Cole A.A., Kuettner K.E. (1997). Prevalence of degenerative morphological changes in the joints of the lower extremity. Osteoarthr. Cartil..

[23] Mankin H.J., Dorfman H., Lippiello L., Zarins A. (1971). Biochemical and metabolic abnormalities in articular cartilage from osteo-arthritic human hips. II. Correlation of morphology with biochemical and metabolic data. J. Bone Jt. Surg. Am..

